# Co-administration of MDR1 and BCRP or EGFR/PI3K inhibitors overcomes lenvatinib resistance in hepatocellular carcinoma

**DOI:** 10.3389/fonc.2022.944537

**Published:** 2022-09-08

**Authors:** Dawei Sun, Juan Liu, Yunfang Wang, Jiahong Dong

**Affiliations:** ^1^ Department of Hepatobiliary and Pancreatic Surgery, The First Hospital of Jilin University, Changchun, China; ^2^ Hepato-Pancreato-Biliary Centre, Beijing Tsinghua Changgung Hospital, Tsinghua University, Beijing, China; ^3^ Research Unit of Precision Hepatobiliary Surgery Paradigm, Chinese Academy of Medical Sciences, Beijing, China

**Keywords:** hepatocellular carcinoma (HCC), lenvatinib resistance (LR), multidrug resistance protein 1 (MDR1), breast cancer resistance protein (BCRP), epidermal growth factor receptor (EGFR), elacridar, gefitinib, copanlisib

## Abstract

Lenvatinib is the first-line treatment for hepatocellular carcinoma (HCC), the most common type of primary liver cancer; however, some patients become refractory to lenvatinib. The underlying mechanism of lenvatinib resistance (LR) in patients with advanced HCC remains unclear. We focused on exploring the potential mechanism of LR and novel treatments of lenvatinib-resistant HCC. In particular, we established a Huh7 LR cell line and performed *in vitro*, bioinformatic, and biochemical assays. Additionally, we used a Huh7-LR cell-derived xenograft mouse model to confirm the results *in vivo*. Following LR induction, multidrug resistance protein 1 (MDR1) and breast cancer resistance protein (BCRP) transporters were markedly upregulated, and the epidermal growth factor receptor (EGFR), MEK/ERK, and PI3K/AKT pathways were activated. *In vitro*, the co-administration of elacridar, a dual MDR1 and BCRP inhibitor, with lenvatinib inhibited proliferation and induced apoptosis of LR cells. These effects might be due to inhibiting cancer stem-like cells (CSCs) properties, by decreasing colony formation and downregulating CD133, EpCAM, SOX-9, and c-Myc expression. Moreover, the co-administration of gefitinib, an EGFR inhibitor, with lenvatinib retarded proliferation and induced apoptosis of LR cells. These similar effects might be caused by the inhibition of EGFR-mediated MEK/ERK and PI3K/AKT pathway activation. *In vivo*, co-administration of lenvatinib with elacridar or gefitinib suppressed tumour growth and angiogenesis. Therefore, inhibiting MDR1 and BCRP transporters or targeting the EGFR/PI3K pathway might overcome LR in HCC. Notably, lenvatinib should be used to treat HCC after LR induction owing to its role in inhibiting tumour proliferation and angiogenesis. Our findings could help develop novel and effective treatment strategies for HCC.

## 1 Introduction

Primary liver cancer (PLC) poses a global health challenge. According to GLOBOCAN 2020, PLC ranks sixth in cancer incidence and second in cancer-related mortality, with approximately 906,000 new cases and 830,000 deaths worldwide in 2020 (1). Unfortunately, these numbers will continue to rise, as over one million individuals will be diagnosed with HCC annually by 2025 (2). Hepatocellular carcinoma (HCC) is the most common form of PLC, accounting for 75–85% of PLC cases (1). Marked improvements have been achieved in the early detection and subsequent treatment of HCC; however, the reality of HCC management remains poor. Presently, only 44% of HCC cases are diagnosed at the localised stage, and 27% and 18% of HCC cases are diagnosed at the regional and distant stages, respectively (3). Consequently, the five-year survival rate of all HCC stages is barely 20%, and this rate decreases to as low as 3% for distant stage HCC (3). The mainstay treatments for localised stage HCC include resection, transplantation, and ablation. However, the presence of underlying diseases (e.g., liver cirrhosis) often complicates surgical management, as liver transplantation is not always available due to the scarcity of donor organs, and local ablation is sometimes not amenable in cases of knotty tumours.

Nevertheless, systemic therapies, such as tyrosine kinase inhibitors (TKIs), provide hope for patients with unresectable HCC and increase overall survival and improve the quality of life of this population (2). Lenvatinib, an oral inhibitor of multiple receptor tyrosine kinases (RTKs), exerts its antitumour effect by inhibiting vascular endothelial growth factor receptors 1–3 (VEGFR1–3), platelet-derived growth factor receptor α (PDGFRα), fibroblast growth factor receptors 1–4 (FGFR1–4), c-KIT, and RET (4). In patients with unresectable HCC, lenvatinib showed non-inferiority in improving survival outcomes compared with sorafenib (5). In the past decade, sorafenib has become the only effective therapeutic choice for patients with advanced HCC, and lenvatinib has been approved as the first-line drug and is used worldwide (6).

Numerous clinical trials have verified the therapeutic efficacy of lenvatinib in patients with HCC. However, the clinical benefits of lenvatinib administration are limited, as some HCCs become refractory to lenvatinib treatment. Hence, substantial interest has focused on the mechanisms of lenvatinib resistance (LR). Particularly, LR is mediated by hepatocyte growth factor/c-MET axis-associated mitogen-activated protein kinase (MAPK)/extracellular signal-regulated kinase (ERK) and phosphatidylinositol 3-kinase (PI3K)/AKT pathway activation (7), upregulated interferon regulatory factor 2 (IRF2) and β-catenin expression (8), FGFR1 overexpression and downstream AKT/mTOR and ERK signalling activation (9), and upregulated VEGFR2 expression and downstream RAS/MEK/ERK pathway activation (10). However, we could hardly find studies on the underlying mechanism of LR following long-term exposure to lenvatinib. Notably, a well-designed combined therapy might successfully inhibit compensatory signalling activation following LR induction; however, a feasible drug combination that could overcome LR has not yet been established.

In this study, we aimed to establish a Huh7 LR cell line to elucidate the underlying mechanism of LR and explore novel drugs that could be used to overcome LR in HCC.

## 2 Materials and methods

### 2.1 Reagents and antibodies

Lenvatinib (*HY-10981*), gefitinib (*HY-50895*), and copanlisib (*HY-15346A*) were purchased from MedChemExpress (Shanghai, China), and elacridar (*S7772*) was purchased from Selleck Chemicals (Shanghai, China). Stock solutions of 20 mM lenvatinib, 100 mM elacridar, and 20 mM gefitinib were dissolved in 100% dimethyl sulfoxide (DMSO), and the stock solution of 10 mM copanlisib was dissolved in Milli-Q water. Antibodies against total epidermal growth factor receptor (EGFR; A11577, ABclonal), phospho-EGFR (AP0820, ABclonal), total PI3K (ab32089, Abcam), phospho-PI3K (4228, CST), total AKT (9272, CST), phospho-AKT (4060, CST), total MEK1/2 (A4868, ABclonal), phospho-MEK1/2 (AP0209, ABclonal), total ERK1/2 (4695, CST), phospho-ERK1/2 (4376, CST), caspase-3 (T40051, Abmart), Bcl-2-associated X (Bax; T40044, Abmart), multidrug resistance protein 1 (MDR1; 13978, CST), breast cancer resistance protein (BCRP; 130244, Abcam), and glyceraldehyde 3-phosphate dehydrogenase (GAPDH; 5174, CST) were used. Horseradish peroxidase (HRP)-conjugated goat anti-rabbit and goat anti-mouse antibodies were purchased from Beyotime Biotechnology (Shanghai, China). Alexa Fluor-conjugated goat anti-rabbit (647 nm*)* and goat anti-mouse (488 nm) antibodies were purchased from Invitrogen (Shanghai, China).

### 2.2 Cell line and cell culture

The Huh7 parental (Huh7 P) cell line was obtained from the Cell Bank of National Biomedicine Research (Beijing, China) and cultivated in Dulbecco’s modified Eagle’s medium (DMEM) supplemented with 10% foetal bovine serum and 1% antibiotics (penicillin and streptomycin) at 37°C and 5% CO_2_. To generate the Huh7 LR cell line, Huh7 P cells were exposed to lenvatinib at an initial dose of 1 μM. Thereafter, the stable cell line was exposed to a lenvatinib concentration that was gradually increased by 1.0–2.0 μM per week. Approximately 10 months later, the Huh7 LR cell line was established and maintained in culture medium containing 20 μM lenvatinib.

### 2.3 RNA sequencing assay

Total RNA was extracted using TRIzol from a 10 cm cell culture plate when the cells reached 70–80% confluence. Three independent samples from each group (Huh7 P and Huh7 LR) were used for RNA-seq by Biomarker Technologies (Beijing, China). Log_2_ (mRNA fold change) was used to assess differentially expressed mRNAs, with the calculated value of *<* -1 or *>* 1 deemed statistically significant (p < 0.001). The online bioinformatics database (DAVID Bioinformatics Resources 6.8, NIAID/NIH; website, https://david.ncifcrf.gov/tools.jsp) was used to analyse the Kyoto Encyclopedia of Genes and Genomes (KEGG) pathways and biological processes based on the RNA-seq results.

### 2.4 Cell proliferation assay

Cells were plated at a density of 4,000 cells per well in a 96-well plate and cultivated overnight. The cells were then exposed to drugs suspended in DMEM (10% FBS) for 96 h. Thereafter, 3-(4,5-dimethylthiazol-2-yl)-2,5-diphenyltetrazolium bromide (MTT) solution at a working concentration of 5 mg/mL was added to the culture medium. After 4 h of incubation, the upper medium was removed, and 100 μL of DMSO was added to dissolve the crystals formed in the lower medium. After 10 min of incubation and shaking, the absorbance was measured at a wavelength of 490 nm. A real-time cell analyser (RTCA) S16 *(*Celligence, China*)* was used to compare cell proliferation ability. During this process, 4,000 cells per well were seeded in a 16-well plate and cultivated in medium containing 20 μM lenvatinib. During the next 72 h, a detector connected to a computer constantly calculated and displayed relative cell proliferation by measuring the electrical resistance of the plate bottom.

### 2.5 Clonogenicity assay

To compare the clonogenicity of Huh7 P and Huh7 LR cell lines, 1,000 cells per well were seeded in a 6-well plate and continuously exposed to culture medium containing lenvatinib (20 μM) for two weeks. To assess the clonogenicity of the Huh7 LR cell line after different drug treatments, 2,000 cells were seeded per well in a 6-well plate, drug-containing media was removed from the cells after 72 h of exposure, and the medium without drugs was changed every 3 days for the next 11 days. After fixing in methyl alcohol for 15 min and staining with crystal violet for 20 min, the colonies were photographed using a camera and analysed using Image J Software.

### 2.6 Cell apoptosis assay

Cells were seeded at a density of 2×10^5^ cells per well in a 6-well plate and cultivated overnight. The cells were then treated with the control or drug-containing media for 72 h. Subsequently, the cells were collected and stained with Annexin V-FITC and propidium iodide (Beyotime Biotechnology, China) for 20 min and then analysed using flow cytometry (Beckman Coulter, USA). At least 5×10^4^ cells were analysed for each sample.

### 2.7 Quantitative real-time polymerase chain reaction

Total RNA was extracted from Huh7 P and Huh7 LR cells using TRIzol reagent, and cDNA synthesis was conducted using a reverse transcription kit (Toyobo FSQ 301, Japan) following the manufacturer’s protocol. Subsequently, qRT-PCR was performed in a total volume of 20 μL, containing Milli-Q water (2 μL), c-DNA (6 μL), forward and reverse primers (2 μL), and q-PCR mix (10 μL; Toyobo QPS-201, Japan). The primers used in this study were manufactured by Ruibiotech (Beijing, China) with the following sequences: β-actin forward: 5′-ATCGTCCACCGCAAATGCTTCTA-3′ and reverse: 5′-AGCCATGCCAATCTCATCTTGTT-3′, MDR1 forward: 5′-GGGAGCTTAACACCCGACTTA-3′ and reverse: 5′-GCCAAAATCACAAGGGTTAGCTT-3′, and BCRP forward: 5′-GCCACAGAGATCATAGAGCCT-3′ and reverse: 5′-TCACCCCCGGAAAGTTGATG-3′. The results were normalised to β-actin expression and are presented as relative mRNA expression levels.

### 2.8 Immunofluorescence staining

Cells (Huh7 P and Huh7 LR) were seeded at a density of 30,000 cells per well in an 8-well plate (BD Falcon 354108, USA) overnight. The cells were then washed thrice with PBS, fixed with 4% paraformaldehyde (PFA) for 20 min, blocked with 10% goat serum, and incubated with primary antibodies MDR1 (Rabbit mAb #13978 CST) and BCRP (Mouse mAb #130244 Abcam) for another day. After washing thrice with PBS, the cells were incubated with conjugated secondary antibodies for two hours at room temperature. Subsequently, 4′,6-diamidino-2-phenylindole was added and incubated for 15 min, and images were captured using a VS200 SlideView (Olympus, Japan).

### 2.9 Western blotting analysis

After incubation with different drugs, the cells were collected and lysed using radioimmunoprecipitation assay buffer supplemented with a protease and phosphatase inhibitor cocktail (Beyotime Biotechnology, China). Equal amounts of protein from each sample were loaded on 8% or 10% sodium dodecyl-sulphate-polyacrylamide gel electrophoresis and transferred onto an Immobilon^®^-P Transfer membrane (Merck Millipore Ltd.). After blocking with 5% non-fat milk, the labelled membrane was incubated with relevant primary antibodies at 4°C overnight. The membranes were then incubated with HRP-conjugated secondary antibodies for two hours at room temperature. Finally, the membranes were incubated with enhanced chemiluminescence reagent (Applygen, China), and the bands were detected. GAPDH was used as an internal reference.

### 2.10 Xenograft tumour in nude mice

BALB/C nude mice (male, 6 weeks old) were obtained from Charles River (Beijing, China). Huh7 LR cells (1.0×10^7^ cells per mouse) were injected into the flanks of the mice. After tumour establishment, the mice were randomly assigned to six groups (five mice per group): the vehicle, lenvatinib (5 mg/kg), gefitinib (80 mg/kg), elacridar (80 mg/kg), lenvatinib (5 mg/kg) combined with elacridar (80 mg/kg), and lenvatinib (5 mg/kg) combined with gefitinib (80 mg/kg) groups. The drugs were suspended in 5‰ carboxymethylcellulose sodium (powder dissolved in Milli-Q water). In the lenvatinib and elacridar group, elacridar was administered two hours prior to lenvatinib. All indicated treatments were orally administered to the mice 5 days per week. Tumour length and width were measured using callipers, and their volumes were calculated using the following formula: tumour volume = ½ length × width^2^. All animal experiments were conducted in accordance with the approved protocol from Charles River (No. P2021049).

### 2.11 Histological analysis

Harvested tumours were fixed in 4% PFA, dehydrated gradually, embedded in paraffin, and sliced into 4 μm thick sections. Some sections were subjected to haematoxylin-eosin (H&E) staining, whereas other sections were used for immunohistochemistry (IHC). After routine IHC procedures, the samples were incubated with primary antibodies against Ki67 (14-5698-80, Invitrogen) and proliferating cell nuclear antigen (PCNA; 13110, CST) at 4°C overnight. The samples were then incubated with secondary antibodies using the VECTASTAIN ^®^ Elite ^®^ ABC Universal Kit, Peroxidase (Horse Anti-Mouse/Rabbit IgG; PK-6200, Vector Laboratories, Inc., USA).

### 2.12 Statistical analysis

OriginPro 2021 software was used to perform data analysis. Data are presented as the mean ± standard deviation based on triplicate experiments, and the final results are representative of more than two independent experiments, excluding the xenograft tumour experiment. All *p* values are denoted as significant at *p < 0.05*. Following the Chou-Talalay method (11), CompuSyn software (ComboSyn, Inc., Paramus, NJ, USA) was used to calculate combination index (CI) values. The CI values reflect the interaction between two drugs (CI < 1, synergism; CI = 1, additive effect; CI > 1, antagonism).

## 3 Results

### 3.1 Establishment of a lenvatinib resistant cell line

**Figure 1:**
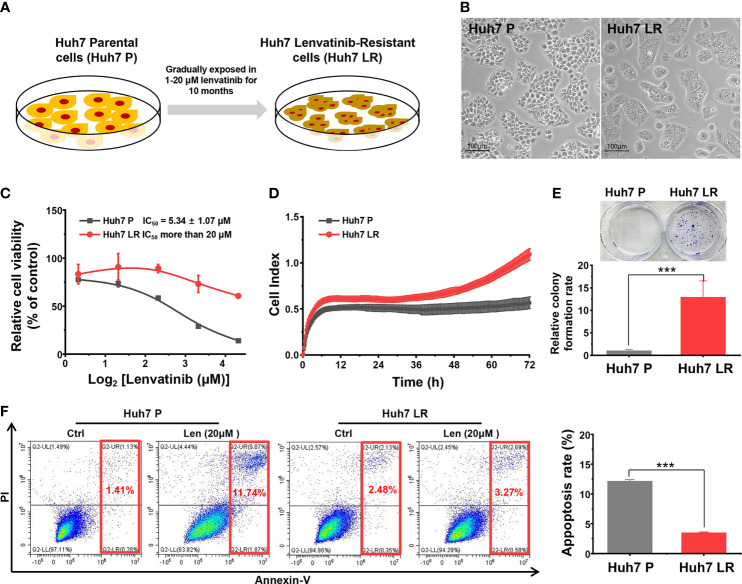
Successfully established Huh7 LR cell line. **(A)** The timeline and lenvatinib concentration. **(B)** The representative morphology of the Huh7 P and Huh7 LR cell lines. **(C)** The MTT assay during 96 h revealed that the Huh7 LR cell line had a substantially higher IC_50_ than the Huh7 P cell line did. **(D)** RTCA revealed that Huh7 LR cells had a higher proliferation rate than Huh7 P cells did in medium containing 20 μM lenvatinib. **(E)** The Huh7 LR cell line exhibited higher colony forming ability than the Huh7 P cell line did in cultivation medium containing 20 μM lenvatinib. **(F)** Flow cytometry assay showed that Huh7 LR cells exhibited higher anti-apoptosis ability than Huh7 P cells did in cultivation medium containing 20 μM lenvatinib for 72 hours. ***p < 0.001.

After continuous exposure to lenvatinib (1–20 μM) for approximately 10 months, the Huh 7 LR cell line was normally passaged and maintained in medium containing 20 μM lenvatinib (**Figure 1A**). In contrast to Huh7 P cells, Huh7 LR cells were smaller in size and grew aggressively (**Figure 1B**). The MTT results revealed that Huh7 LR cells exhibited a higher proliferation rate than Huh7 P cells did in cultivation medium containing different lenvatinib concentrations (1.25, 2.5, 5, 10, and 20 μM), and the half maximal inhibitory concentration (IC_50_) of lenvatinib in Huh7 LR cells (IC_50_ > 20 μM) was significantly higher than that in Huh7 P cells (IC_50_ 5.34 ± 1.07 μM) (**Figure 1C**). Meanwhile, Huh7 LR cells exhibited a relatively higher proliferation rate (**Figure 1D**) and higher colony forming ability (**Figure 1E**) than Huh7 P cells did in medium containing 20 μM lenvatinib. Moreover, Huh7 LR cells exhibited a higher anti-apoptotic activity than Huh7 P cells did in cultivation medium containing 20 μM lenvatinib (**Figure 1F**). These results confirmed that the Huh7 LR cell line was resistant to lenvatinib.

### 3.2 Transcriptomic analysis results

**Figure 2:**
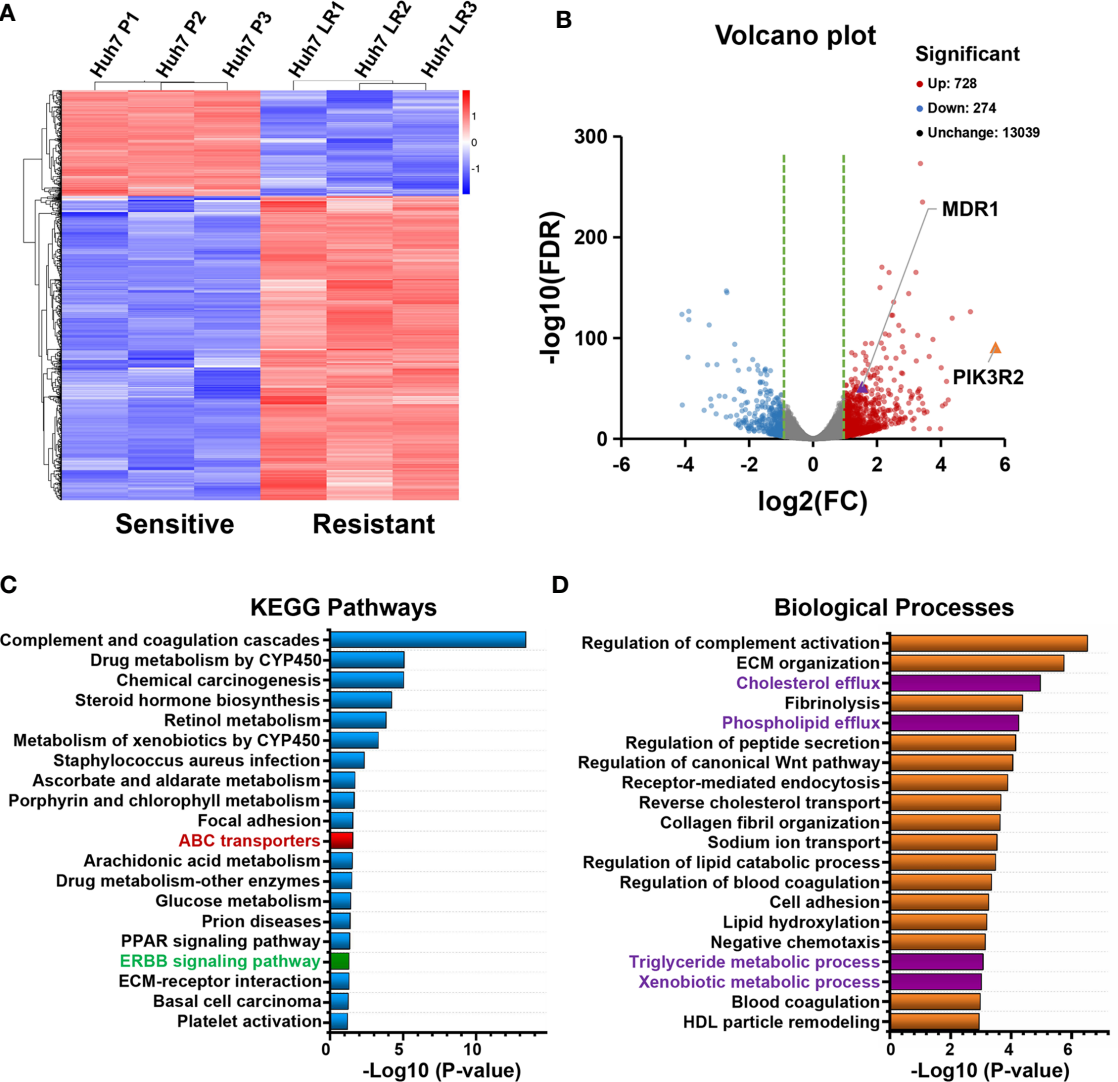
Transcriptomic analysis of Huh7 P and Huh7 LR cells based on RNA sequencing (three samples for each group). **(A, B)** Clustering heatmap and volcano plot show differentially expressed genes between the Huh7 P and Huh7 LR cell lines (> 2-fold change, p < 0.001). **(C, D)** KEGG pathways and biological processes associated with significantly upregulated genes in Huh7 LR cells (> 2-fold change, *p* < 0.001).

RNA-seq results were obtained to assess differentially expressed mRNAs between the Huh7 P and Huh7 LR cell lines (**Supplementary Table 1**). Three independent samples were examined for each cell line (**Figure 2A**), and the Huh7 LR cell line exhibited 728 upregulated and 274 downregulated genes compared with those in the Huh7 P cell line (**Figure 2B**). KEGG pathway enrichment analysis revealed that pathways related to metabolism and ATP-binding cassette (ABC) transporters and the ERBB signalling pathway were enriched after LR induction (**Figure 2C**). Additionally, gene annotation analysis of biological processes demonstrated that cellular efflux and metabolic processes were increased after LR induction (**Figure 2D**).

### 3.3 MDR1 and BCRP overexpression and EGFR signalling pathway activation following LR induction

**Figure 3:**
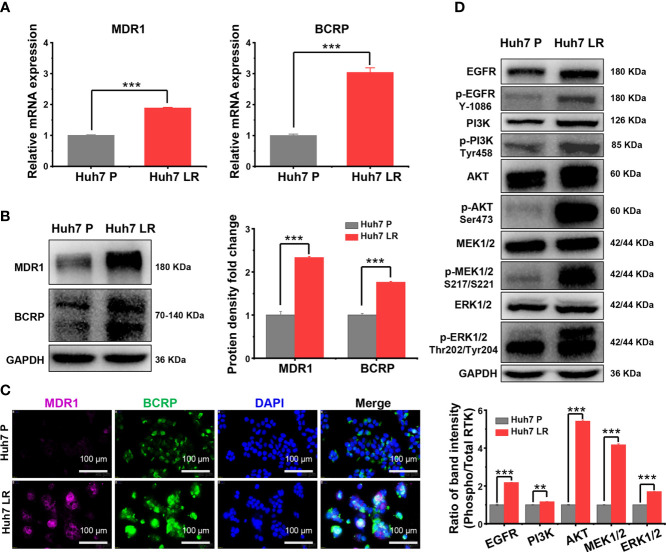
MDR1 and BCRP overexpression and EGFR signalling pathway activation following LR induction. **(A–C)** qRT-PCR, western blotting, and immunofluorescence analysis demonstrated that MDR1 and BCRP expression was upregulated following LR induction. Scale bars, 100 μm. **(D)** Western blotting revealed that EGFR and its downstream MEK/ERK and PI3K/AKT pathways were markedly activated following LR induction. **p < 0.01. ***p < 0.001.

The expression of MDR1 and BCRP, important ATP-binding cassette (ABC) transporters, was upregulated according to the RNA-seq results (**Supplementary Table 2**). We used qRT-PCR, western blotting, and immunofluorescence staining to further verify MDR1 and BCRP expression levels. First, the qRT-PCR results revealed that Huh7 LR cells exhibited significantly higher MDR1 and BCRP mRNA levels than Huh7 P cells did (**Figure 3A**). Second, western blotting demonstrated that Huh7 LR cells exhibited significantly higher MDR1 and BCRP levels than Huh7 P cells did (**Figure 3B**). Third, immunofluorescence staining revealed that MDR1 and BCRP were located in the cell membrane, and their expression was significantly higher in Huh7 LR cells than in Huh7 P cells (**Figure 3C**).

The EGFR signalling pathway is an important branch of the ERBB signalling pathway, and the activation of EGFR signalling pathway is a hallmark of human malignancies (12–14). **Supplementary Table 3** demonstrates that the transcriptional levels of EGFR and the downstream RAS/RAF/MEK/ERK and PI3K/AKT/mTOR pathways were normal or upregulated after LR induction. Importantly, among the upregulated mRNAs, PIK3R2, an oncogene involved in the physiological activation of PI3K (15), ranked first in fold-change (Log_2_FC = 5.71) (**Supplementary Tables 1**, **2**, **Figure 2B**). However, RNA-seq only reflects transcriptional level but cannot comprehensively reflect protein levels and functional alterations. Therefore, western blotting was performed to determine the levels of total and phosphorylated proteins involved in EGFR signalling and its downstream pathways. The western blot results revealed that phosphorylated EGFR, PI3K, AKT, MEK1/2, and ERK1/2 were significantly upregulated in Huh7 LR cells compared to those in Huh7 P cells (**Figure 3D**).

### 3.4 *In vitro* antitumour effect of combined treatments

#### 3.4.1 Elacridar ameliorated LR by inhibiting MDR1 and BCRP

**Figure 4:**
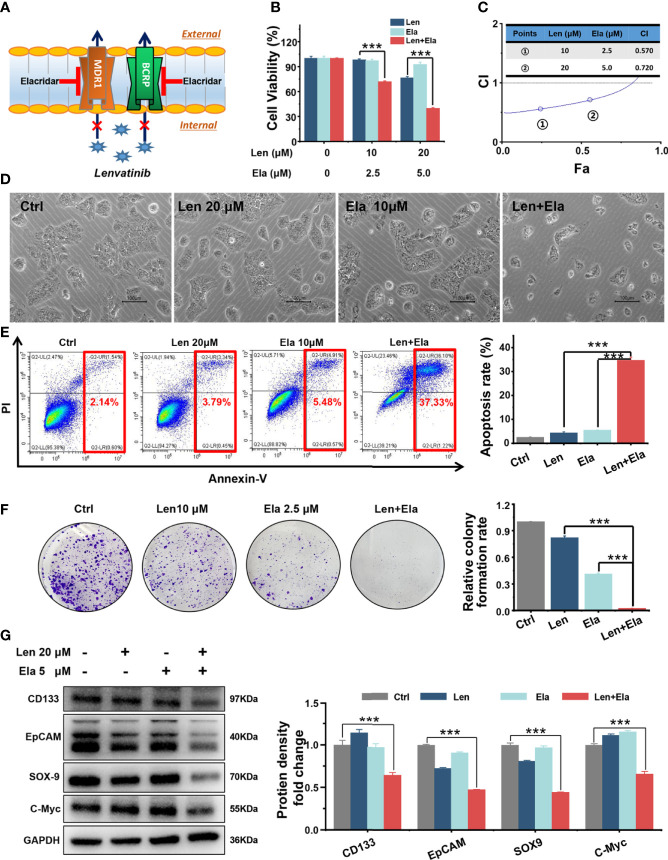
*In vitro* combined antitumour effect of lenvatinib and elacridar. **(A)** Schematic diagram indicates that elacridar dually inhibited MDR1 and BCRP. **(B, C)** The MTT results and CI plots confirmed that elacridar synergised with lenvatinib to inhibit Huh7 LR cell viability. **(D, E)** Co-treatment with elacridar and lenvatinib enhanced cell apoptosis, as shown in micrographs and flow cytometry plots. **(F, G)** Combined elacridar and lenvatinib treatment inhibited colony formation and downregulated CD133, EpCAM, SOX-9, and c-Myc expression. ***p < 0.001.

Both MDR1 and BCRP mediate drug efflux from tumour cells, which decreases the effective concentration of antitumour drugs and results in chemotherapeutic failure (16–18). Here, we speculated that elacridar, a dual MDR1 and BCRP inhibitor (19), could overcome LR in Huh7 LR cells by inhibiting MDR1 and BCRP (**Figure 4A**).

According to the MTT results, the relative cell viability of the 20 μM lenvatinib-treated group was 76.23%, whereas the relative cell viability of the 5 μM elacridar-treated group was 92.49%. However, the relative cell viability of the 20 μM lenvatinib- and 5 μM elacridar-treated group significantly decreased to as low as 39.67% (**Figure 4B**). The synergistic antitumour effect of lenvatinib and elacridar was further verified using CI values and the Chou-Talalay method. As shown in **Figure 4C**, the calculated CI values were < 1, indicating that elacridar synergised with lenvatinib to inhibit Huh7 LR cell proliferation. Thereafter, flow cytometry was performed to assess the pro-apoptotic effect of elacridar in Huh7 LR cells following treatment with a single drug or with co-administration of lenvatinib for 72 h (**Figure 4D**). According to the quantitative results (**Figure 4E**), lenvatinib (20 μM) in combination with elacridar (10 μM) significantly induced Huh7 LR cell apoptosis (apoptosis rate, 37.32%) compared with that of the control (1% DMSO), lenvatinib (20 μM), and elacridar (10 μM) groups (apoptosis rates, 2.14%, 3.79%, and 5.48%, respectively). Therefore, elacridar sensitised Huh7 LR cells to lenvatinib treatment.

Additionally, our *in vitro* results demonstrated that the combination of elacridar with lenvatinib significantly inhibited colony formation (**Figure 4F**) and decreased CD133, epithelial cellular adhesion molecule (EpCAM), SRY-box transcription factor 9 (SOX-9), and c-Myc expression (**Figure 4G**).

#### 3.4.2 Gefitinib or copanlisib ameliorated LR by targeting the EGFR/PI3K pathway

**Figure 5:**
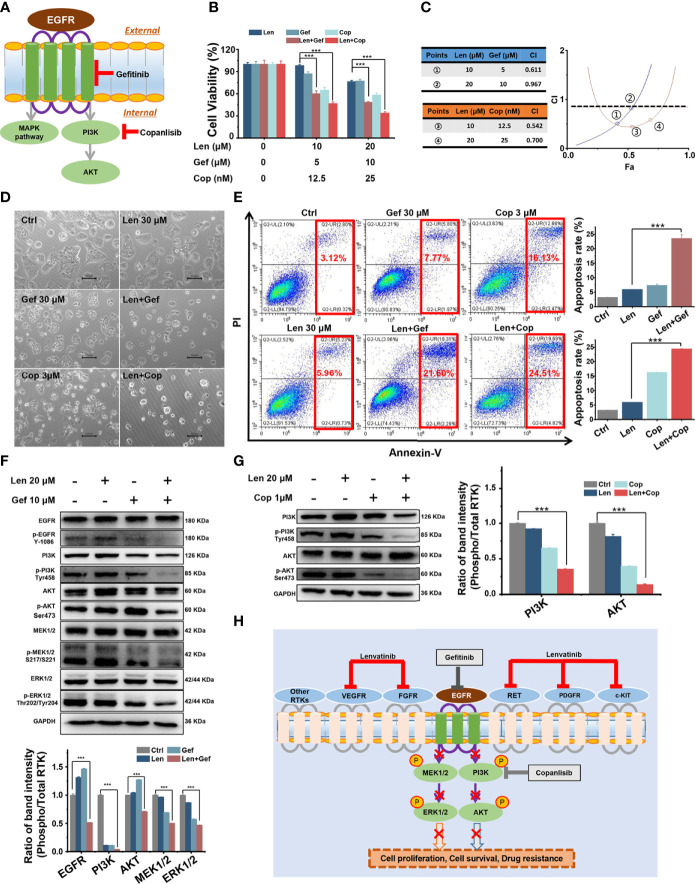
*In vitro* combined antitumour effect of lenvatinib and gefitinib or copanlisib. **(A)** Schematic diagram indicates that gefitinib and copanlisib targeted EGFR and PI3K, respectively. **(B, C)** The MTT results and CI plots confirmed that combined treatments synergistically inhibited cell viability. **(D, E)** The addition of gefitinib or copanlisib enhanced cell apoptosis, as shown in micrographs and flow cytometry plots. **(F, G)** The EGFR pathway was significantly inhibited by the addition of gefitinib, and the PI3K/AKT pathway was significantly inhibited by the addition of copanlisib. **(H)** Schematic diagram delineates the proposed mechanism of overcoming LR in HCC by targeting the EGFR/PI3K pathway. ***p < 0.001.

EGFR and/or PI3K/AKT pathway activation is associated with chemotherapeutic resistance in human cancers (20, 21). Unfortunately, lenvatinib exerts antitumour effects by targeting multiple cell membrane RTKs, including VEGF1-3, PDGFR, FGFR1-4, c-KIT, and RET rather than EGFR (4). Here, we speculated that EGFR signalling pathway activation might be associated with LR; therefore, we investigated whether the addition of TKIs targeting the EGFR/PI3K/AKT pathway could overcome LR. We selected and tested FDA-approved clinical drugs, including gefitinib (targeting EGFR) and copanlisib (targeting PI3K) (**Figure 5A**).

First, we performed an MTT assay to assess the effect of gefitinib or copanlisib on Huh7 LR cell proliferation following treatment with a single drug or with co-administration of lenvatinib. As shown in **Figure 5B**, the addition of gefitinib or copanlisib significantly enhanced the inhibitory effect of lenvatinib in Huh7 LR cells. We used the Chou-Talalay method and determined that lenvatinib and gefitinib or copanlisib synergistically inhibited cell proliferation, as the calculated CI values were < 1 (**Figure 5C**). Thereafter, we performed flow cytometry to assess the pro-apoptotic effect of gefitinib or copanlisib in Huh7 LR cells after drug treatment for 72 h (**Figure 5D**). According to the quantitative results (**Figure 5E**), the addition of gefitinib or copanlisib significantly enhanced the pro-apoptotic effect of lenvatinib in Huh7 LR cells. Therefore, gefitinib or copanlisib sensitised Huh7 LR cells to lenvatinib treatment.

Regarding the potential antitumour mechanism, western blotting revealed that co-treatment with gefitinib and lenvatinib significantly inhibited the phosphorylation of EGFR, PI3K, AKT, MEK1/2, and ERK1/2 (**Figure 5F**), whereas the combination of copanlisib and lenvatinib significantly inhibited the phosphorylation of PI3K and AKT (**Figure 5G**). Moreover, the addition of gefitinib or copanlisib increased the levels of apoptosis-associated proteins, including caspase-3 and Bax (**Supplementary Figure 1**). Here, we proposed that upon targeting cell membrane RTKs, including VEGFR, FGFR, RET, PDGFR, and c-KIT, with lenvatinib, EGFR was activated to compensate for LR. The downstream MEK/ERK and PI3K/AKT pathways were then activated in response to EGFR activation, which resulted in LR by promoting cell proliferation and survival. However, upon targeting EGFR with gefitinib or PI3K with copanlisib in combination with lenvatinib, compensatory activation of the EGFR signalling pathway or its downstream PI3K/AKT pathway, respectively, was inhibited (**Figure 5H**).

### 3.5 *In vivo* antitumour effect of combined treatments

**Figure 6:**
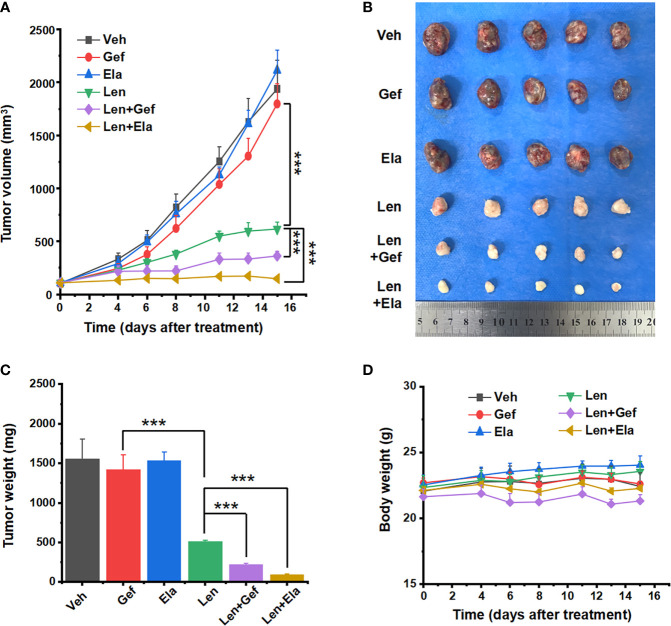
Lenvatinib in combination with elacridar or gefitinib suppressed HCC xenograft growth. **(A)** Xenograft response to treatment with vehicle, elacridar (80 mg/kg), gefitinib (80 mg/kg), lenvatinib (5 mg/kg), and drug combination (elacridar 80 mg/kg and lenvatinib 5 mg/kg or gefitinib 80 mg/kg and lenvatinib 5 mg/kg). **(B)** Harvested tumours are arranged according to the treatment group. **(C)** Tumour weights were measured after resection. **(D)** Mouse body weights were measured during the treatment. ***p < 0.001.

The *in vivo* antitumour effects of lenvatinib in combination with elacridar or gefitinib were assessed using xenografts derived from the Huh7 LR cell line. One week after tumour cell injection, the average xenograft size reached approximately 6 mm in diameter, and therapeutic treatment was initiated accordingly. Subsequently, the tumour volume and mouse body weight were measured once every two to three days. The mice were orally administered with the drugs for two weeks and then sacrificed. The harvested tumours were imaged and their corresponding weights were measured. Compared with that in the vehicle group, neither elacridar nor gefitinib inhibited tumour growth, and lenvatinib-based co-treatments significantly suppressed tumour growth (**Figures 6A–C**). Intriguingly, lenvatinib-based co-treatments exerted a much better antitumour effect than lenvatinib treatment alone did. Particularly, the co-administration of lenvatinib with elacridar exhibited the most potent antitumour efficacy (**Figures 6A–C**). During the treatment, no significant side effects were observed, as the mouse body weights were comparable in different groups (**Figure 6D**).

**Figure 7:**
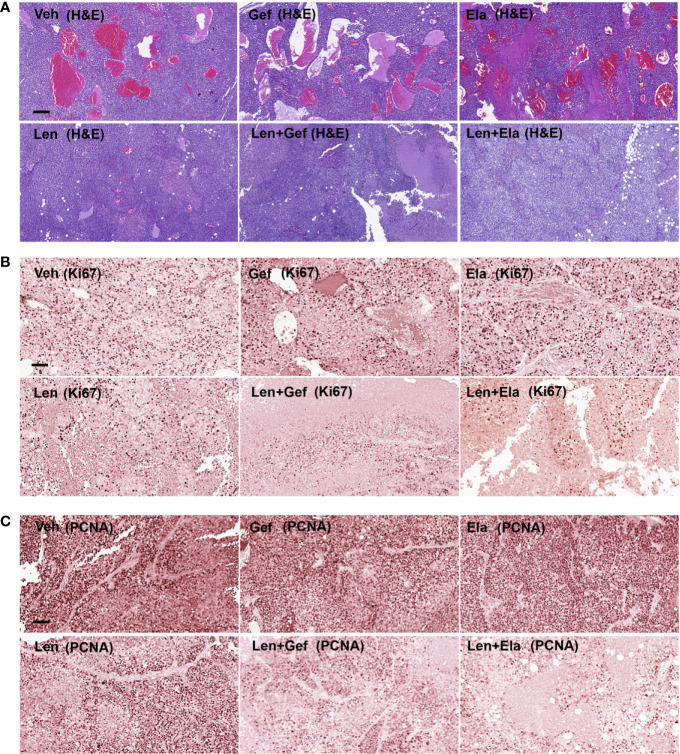
Lenvatinib in combination with elacridar or gefitinib inhibited tumour proliferation and angiogenesis. **(A)** Representative images of blood vessel density visualised by H&E staining. Scale bars, 200 μM. **(B, C)** IHC for Ki67 and PCNA expression. Scale bars, 100 μM.

Regarding the histological analysis, lenvatinib alone and lenvatinib-based co-administrations significantly inhibited tumour angiogenesis, which could be easily determined by observing the general shape of the harvested tumours (**Figure 6B**). Upon further analysis using pathological H&E staining, remaining tumour micro-vessels were observed in the group treated with lenvatinib alone, but not in the group treated with co-administration of gefitinib or co-administration of elacridar (**Figure 7A**). Lenvatinib alone could inhibit cell proliferation; however, lenvatinib-based co-administration enhanced the inhibition of cell proliferation, which was assessed using IHC for Ki67 and PCNA (**Figures 7B, C**).

## 4 Discussion

Systemic therapies for unresectable HCC are limited. In addition to sorafenib, lenvatinib is currently the first-line treatment for patients with advanced HCC worldwide (6). Recently, a meta-analysis comprising five clinical studies with 1,481 patients demonstrated that lenvatinib treatment significantly improved progression-free survival (PFS), objective response rate (ORR), and disease control rate compared with those of sorafenib treatment in patients with advanced HCC (22). However, a standard salvage treatment has not yet been established for patients with advanced HCC after lenvatinib therapy failure. Considering the dismal outcomes of patients with HCC after lenvatinib treatment failure, exploring the underlying mechanism of LR and novel drugs to overcome LR is warranted.

To the best of our knowledge, this is the first study to reveal that ABC transporters and the EGFR signalling pathway are activated in HCC after long-term exposure to lenvatinib. MDR1 or P-glycoprotein and BCRP, important ABC transporters, have consistently been implicated in mediating multiple drug resistance by promoting drug efflux in various human cancers (16–18). Coincidentally, lenvatinib is a substrate for MDR1 (23, 24); however, the changes in MDR1 and BCRP transporters after LR induction have not yet been clarified. Moreover, EGFR, a pioneer member of the RTK family, is frequently overexpressed in human cancers (13, 25, 26), and its activation is crucial for essential cancer cell processes, including cell growth, survival, and drug resistance (25). Unfortunately, lenvatinib targets multiple cell membrane RTKs but EGFR (4). Recently, one study has revealed that blocking EGFR by gefitinib and lenvatinib exhibited a relatively potent antitumour efficacy in HCC (27), whereas the activation status of EGFR and its downstream pathways (MEK/ERK and PI3K/AKT) after LR induction in HCC has not been fully understood. Notably, our *in vitro* results revealed that MDR1 and BCRP transporters were significantly upregulated, and EGFR and the MEK/ERK and PI3K/AKT pathways were activated after LR induction.

Subsequently, considering that ABC transporters and EGFR signalling pathways were activated after LR induction, we utilised three drugs: elacridar, gefitinib, and copanlisib. Elacridar (GF12098) is a dual MDR1 and BCRP inhibitor (19). *In vitro*, preclinical, and clinical studies have demonstrated that co-administration of elacridar could reverse MDR1 and/or BCRP-mediated chemotherapeutic resistance and increase systemic exposure to antitumour drugs by inhibiting efflux pumps (19, 28, 29). Furthermore, gefitinib selectively inhibits EGFR and was first used to treat advanced non-small cell lung cancer after other treatments failed (30). As monotherapy or combination therapy, gefitinib is also used to treat other human malignancies (31). Moreover, gefitinib inhibits the growth and accelerates the apoptosis of human HCC cells and promotes cell cycle arrest in these cells (32). Blocking EGFR by gefitinib exerts antitumour effects by reducing HCC nodule formation in rats (33). Lastly, copanlisib (BAY80-6946) has emerged as a newly developed pan-PI3K inhibitor (34, 35) and was first approved for treating relapsed follicular lymphoma (36). Subsequently, copanlisib has been used in patients with advanced or refractory solid tumours (37). *In vitro* studies have recently demonstrated that copanlisib synergises with sorafenib to promote cell death in HCC (38). However, the therapeutic role of these drugs in HCC after LR induction has not been reported.

Previous *in vivo* studies have demonstrated that lenvatinib is a substrate of MDR1, and inhibiting MDR1 using rifampicin or ketoconazole can significantly increase plasma lenvatinib concentrations in healthy adults (23, 24). Elacridar, a third-generation MDR1 inhibitor and a dual inhibitor of MDR1 and BCRP transporters (39), can improve therapeutic efficacy in various diseases by blocking drug efflux, according to previous *in vitro*, preclinical, and clinical studies (28). Theoretically, elacridar should also inhibit lenvatinib efflux by inhibiting MDR1 and BCRP efflux pumps. Additionally, cancer stem-like cells (CSCs) harbouring stem cell-like properties, including aberrant differentiation and self-renewal potential, are associated with chemotherapeutic resistance in cancers (40–42). Coincidentally, Sugano et al. found that inhibiting MDR1 using elacridar inhibits CSC properties (43). Parallelly, our *in vitro* experiments demonstrated that inhibiting lenvatinib efflux by inhibiting MDR1 and BCRP efflux pumps might represent the potential mechanism of synergism between elacridar and lenvatinib to overcome LR. Here, we attempted to summarise and explain the antitumour effect of combined treatment. Lenvatinib in combination with elacridar exerted a significantly synergistic antitumour effect *in vitro* and the most significant antitumour effect *in vivo*. Additionally, a combination of lenvatinib and elacridar significantly inhibited CSC properties by decreasing colony formation and downregulating CD133, EpCAM, SOX-9, and c-Myc expression. Moreover, lenvatinib alone suppresses CSCs marked by CD133 and CD44 expression in HCC (44), and this inhibitory effect was presumably enhanced because elacridar could inhibit lenvatinib efflux by inhibiting MDR1 and BCRP transporters, which might account for the above findings. Furthermore, co-administration of lenvatinib and gefitinib significantly inhibited EGFR, MEK/ERK, and PI3K/AKT activation, and co-administration of lenvatinib with copanlisib significantly inhibited PI3K/AKT activation; both combinations exerted synergistic antitumour effects *in vitro*. Moreover, gefitinib in combination with lenvatinib exerted potent antitumour effects *in vivo*.

According to our literature review, other research groups are also attempting to develop salvage systemic treatment for patients with HCC after lenvatinib treatment failure. For example, one clinical study of 22 participants with failed lenvatinib therapy who received second-line regorafenib treatment revealed that the PFS and ORR were 3.2 (range, 1.5–4.9) months and 13.6%, respectively (45). Another clinical study involving 13 patients with unresectable HCC who were treated with sorafenib after lenvatinib treatment failure revealed that the PFS and ORR were 4.1 (range, 2.1–9.2) months and 15.3% (2/13), respectively (46). The survival outcomes were poor in patients who received second-line treatments after lenvatinib withdrawal. Coincidentally, our study found that xenografts grew faster and exhibited increased angiogenesis in the groups without lenvatinib treatment, including the gefitinib-treated group.

We proposed hypotheses regarding the dismal patient outcomes after stopping lenvatinib treatment and the increased xenograft growth observed in the groups without lenvatinib treatment. One hypothesis is that despite drug resistance, lenvatinib still blocked the intracellular signal transduction phosphorylation cascade by inhibiting ligand binding to cell membrane RTKs; in particular, the activation of VEGFR correlated with angiogenesis and the activation of PDGFR, FGFR, c-KIT, and RET correlated with cell proliferation (47, 48). Another hypothesis is that lenvatinib inhibited CSCs harbouring stem cell-like properties, including aberrant differentiation and self-renewal potential, which was verified by our *in vitro* experiments (lenvatinib inhibited colony formation) and the findings reported by Shigesawa et al. (lenvatinib inhibited CD133- and CD44-positive CSCs) (44). However, once lenvatinib is withdrawn, the underlying inhibition of intracellular signal transduction and of CSC-associated characteristics is reversed, which consequently accelerates tumour growth and angiogenesis. Therefore, we wondered whether patient outcomes would improve if lenvatinib was continuously administered in combination with second-line treatment after LR, which is merely our theoretical conjecture based on the xenograft experiment results. Certain issues, particularly the side effects and energy expenditure caused by combination treatment, remain to be seriously considered.

Finally, our study had certain limitations and provided scope for further research. First, our *in vitro* and *in vivo* results were based on a Huh7 LR cell line and the Huh7 LR cell line-derived xenografts. Thus, our findings and conclusions should be further replicated and verified in more cell lines, as well as in patients with HCC after LR induction, if possible. Second, some drugs (e.g., lenvatinib and gefitinib) used in this study are readily soluble in DMSO but not in cultivation media, which resulted in the parallelism of MTT results being lower than expected. Third, the antitumour effect of lenvatinib combined with copanlisib was not examined in the xenograft model. Therefore, the antitumour effects of copanlisib, an FDA-approved drug, alone or in combination with lenvatinib in HCC after LR induction should be investigated. Lastly, *in vivo* side effects of drug combinations, such as changes in organ function and/or microscopic structure, should also be assessed in the future.

## 5 Conclusions

In summary, inhibiting MDR1 and BCRP transporters or targeting the EGFR/PI3K pathway might overcome LR in HCC. Intriguingly, we observed the synergistic effects of lenvatinib and elacridar or gefitinib. Notably, lenvatinib should be used to treat HCC after LR because of its role in inhibiting tumour proliferation and angiogenesis. Importantly, our results and raised hypotheses should be further evaluated in patients with HCC following LR induction. Nevertheless, we provide a theoretical basis for the salvage treatment of HCC after LR induction.

## Data availability statement

The original contributions presented in the study are publicly available. This data can be found at https://www.ncbi.nlm.nih.gov/geo/, using the following accession numbers: GSE211850, GSM6503394, GSM6503395, GSM6503396, GSM6503397, GSM6503398, GSM6503399.

## Ethics statement

The animal study in this research was reviewed and approved by Charles River of Beijing (No. P2021049).

## Author contributions

JL, YW, and JD designed and supervised this research. DS performed the experiments, analysed the data, and wrote this paper. All authors contributed to the article and approved the submitted version.

## Funding

This work was supported by the National Natural Science Foundations of China (81730052, 81930119, 82090051, 82090053, and 32000970), Chinese Academy of Medical Sciences Innovation Fund for Medical Sciences (2019-I2M-5-056), Natural Science Foundation of Beijing (7214306), Beijing Hospitals Authority, Ascent Plan (DFL20190901), and Beijing Hospitals Authority Youth Programme (QML20200903).

## Conflict of interest

The authors declare that the research was conducted in the absence of any commercial or financial relationships that could be construed as a potential conflict of interest.

## Publisher’s note

All claims expressed in this article are solely those of the authors and do not necessarily represent those of their affiliated organizations, or those of the publisher, the editors and the reviewers. Any product that may be evaluated in this article, or claim that may be made by its manufacturer, is not guaranteed or endorsed by the publisher.
