# Supplementary material for: Co-administration of MDR1 and BCRP or EGFR/PI3K inhibitors overcomes lenvatinib resistance in hepatocellular carcinoma
Source: Front Oncol. 2022 Sep 8;12:944537. doi: 10.3389/fonc.2022.944537 (PMC9496645; doi:10.3389/fonc.2022.944537)
Supplement: Supplementary file 1 [file DataSheet_1.zip › Supplementary Materials/Supplementary Figure 1.docx]

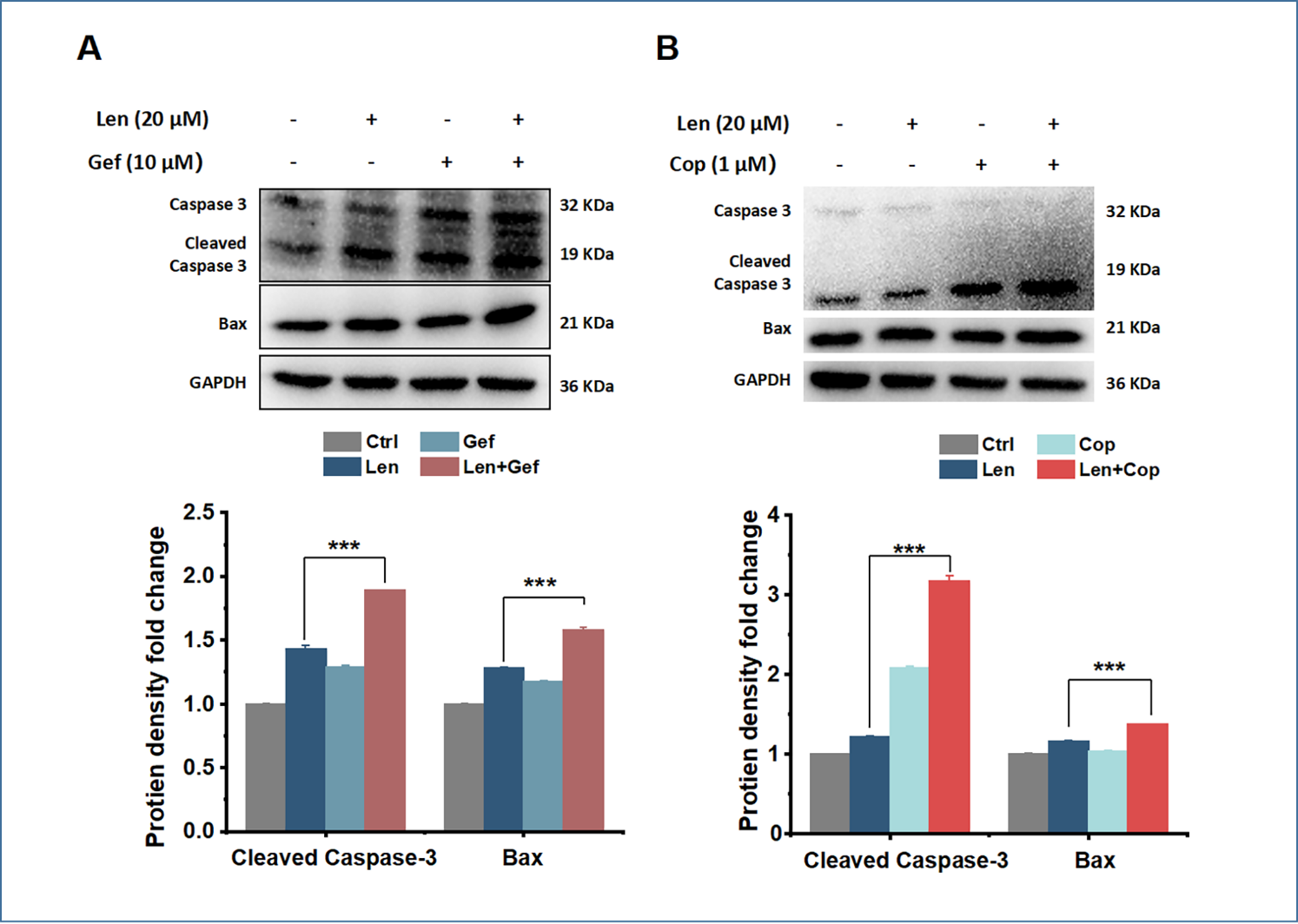


**Supplementary Figure 1.** Combined lenvatinib and gefitinib significantly enhanced cellular apoptosis associated proteins generation (**A**). Combined lenvatinib and copanlisib significantly enhanced cellular apoptosis associated proteins generation (**B**).
