# Supplementary material for: Co-administration of MDR1 and BCRP or EGFR/PI3K inhibitors overcomes lenvatinib resistance in hepatocellular carcinoma
Source: Front Oncol. 2022 Sep 8;12:944537. doi: 10.3389/fonc.2022.944537 (PMC9496645; doi:10.3389/fonc.2022.944537)
Supplement: Supplementary file 1 [file DataSheet_1.zip › Supplementary Materials/Supplementary Table 2.docx]

**Supplementary Table 2.** Selected gene alterations (*FPKM*) indicative of ABC transporters between Huh7 P and Huh7 LR by RNA-sequence results.

| **Gen ID** | **Gene name** | **Huh7 P1** | **Huh7 P2** | **Huh7 P3** | **Huh7 LR1** | **Huh7 LR2** | **Huh7 LR3** | **Regulation** |
| --- | --- | --- | --- | --- | --- | --- | --- | --- |
| *ENSG00000085563* | ***MDR1*** | 11.758198 | 12.806151 | 11.530556 | 30.189118 | 38.408862 | 39.0556 | Up |
| *ENSG00000005471* | ***ABCB4*** | 2.819034 | 3.100753 | 2.766906 | 4.730745 | 3.58348 | 4.298298 | Normal |
| *ENSG00000004846* | ***ABCB5*** | 0.031784 | 0.027102 | 0.004791 | 0.02853 | 0.039138 | 0.074767 | Normal |
| *ENSG00000115657* | ***ABCB6*** | 24.404129 | 21.559631 | 24.151605 | 15.519795 | 15.139742 | 14.632465 | Normal |
| *ENSG00000131269* | ***ABCB7*** | 7.278868 | 6.75673 | 6.07249 | 7.270857 | 6.164602 | 7.07946 | Normal |
| *ENSG00000197150* | ***ABCB8*** | 12.379014 | 10.740325 | 10.540573 | 7.140832 | 9.049163 | 8.692858 | Normal |
| *ENSG00000150967* | ***ABCB9*** | 2.403529 | 2.183666 | 2.262676 | 2.377531 | 2.592422 | 2.36564 | Normal |
| *ENSG00000135776* | ***ABCB10*** | 7.120683 | 8.004552 | 7.49216 | 8.255022 | 7.906838 | 8.885207 | Normal |
| *ENSG00000073734* | ***ABCB11*** | 0.178010 | 0.211602 | 0.143486 | 0.141631 | 0.062898 | 0.113695 | Normal |
| *ENSG00000160179* | ***ABCG1*** | 0.098414 | 0.091847 | 0.047803 | 1.009343 | 0.425095 | 0.427819 | Up |
| *ENSG00000118777* | ***BCRP*** | 2.86988 | 2.893925 | 2.664266 | 3.940654 | 3.892161 | 4.081584 | UP |
| *ENSG00000143921* | ***ABCG8*** | 0.014395 | 0.006011 | 0.003874 | 0.00605 | 0.003689 | 0.007624 | Normal |
| *ENSG00000103222* | ***ABCC1*** | 2.325562 | 2.211821 | 1.761181 | 0.662485 | 0.699472 | 0.656555 | Down |
| *ENSG00000023839* | ***ABCC2*** | 39.244137 | 41.040406 | 40.631322 | 33.275312 | 35.47711 | 35.329434 | Normal |
| *ENSG00000108846* | ***ABCC3*** | 10.544092 | 10.411725 | 9.699852 | 17.820575 | 20.12062 | 19.782792 | Normal |
| *ENSG00000125257* | ***ABCC4*** | 12.203005 | 12.464177 | 11.355185 | 13.172315 | 13.189832 | 13.128627 | Normal |
| *ENSG00000114770* | ***ABCC5*** | 7.90255 | 7.391025 | 7.174987 | 6.153524 | 7.129093 | 6.70389 | Normal |
| *ENSG00000091262* | ***ABCC6*** | 10.693769 | 10.812308 | 10.289865 | 13.503664 | 16.659398 | 16.119648 | Normal |
| *ENSG00000124574* | ***ABCC10*** | 3.573112 | 3.470639 | 3.305581 | 3.513207 | 3.795925 | 3.622602 | Normal |
| *ENSG00000121270* | ***ABCC11*** | 0.156036 | 0.085025 | 0.13218 | 0.071784 | 0.066868 | 0.077656 | -- |
