# Supplementary material for: Co-administration of MDR1 and BCRP or EGFR/PI3K inhibitors overcomes lenvatinib resistance in hepatocellular carcinoma
Source: Front Oncol. 2022 Sep 8;12:944537. doi: 10.3389/fonc.2022.944537 (PMC9496645; doi:10.3389/fonc.2022.944537)
Supplement: Supplementary file 1 [file DataSheet_1.zip › Supplementary Materials/Supplementary Table 3.docx]

**Supplementary Table 3.** Available gene alterations (*FPKM*) indicative of EGFR along with its downstream PI3K/AKT and RAS/MEK/ERK pathway between Huh7 P and Huh7 LR by RNA- sequence results.

| ***Gen ID*** | **Gene name** | **Huh7 P1** | **Huh7 P2** | **Huh7 P3** | **Huh7 LR1** | **Huh7 LR2** | **Huh7 LR3** | **Regulation** |
| --- | --- | --- | --- | --- | --- | --- | --- | --- |
| *ENSG00000196455* | ***PIK3R4*** | 12.818208 | 12.315599 | 12.03244 | 12.067729 | 10.655051 | 11.49925 | Normal |
| *ENSG00000117461* | ***PIK3R3*** | 4.132078 | 3.864096 | 3.863041 | 5.312099 | 5.803066 | 5.357492 | Normal |
| *ENSG00000105647* | ***PIK3R2*** | 0.594381 | 0.346615 | 0.30276 | 37.233568 | 59.363496 | 53.321943 | Up |
| *ENSG00000145675* | ***PIK3R1*** | 11.019805 | 11.034615 | 10.564643 | 12.880477 | 10.827822 | 13.135583 | Normal |
| *ENSG00000100100* | ***PIK3IP1*** | 0.837717 | 1.097633 | 0.723382 | 4.854676 | 3.474674 | 3.52392 | Up |
| *ENSG00000171608* | ***PIK3CD*** | 0.297633 | 0.32287 | 0.29026 | 0.51351 | 0.297558 | 0.24593 | Normal |
| *ENSG00000051382* | ***PIK3CB*** | 13.527374 | 12.579467 | 12.250072 | 13.017099 | 11.92948 | 13.021926 | Normal |
| *ENSG00000121879* | ***PIK3CA*** | 4.570342 | 4.237688 | 4.276983 | 4.319934 | 3.454947 | 4.451264 | Normal |
| *ENSG00000078142* | ***PIK3C3*** | 4.427277 | 4.665033 | 4.710515 | 4.271422 | 3.485292 | 4.308668 | Normal |
| *ENSG00000139144* | ***PIK3C2G*** | 0.386511 | 0.509331 | 0.772978 | 0.694382 | 0.425448 | 0.836743 | Normal |
| *ENSG00000133056* | ***PIK3C2B*** | 8.788989 | 7.433921 | 7.004573 | 6.57511 | 6.235904 | 6.488685 | Normal |
| *ENSG00000011405* | ***PIK3C2A*** | 15.228916 | 13.282734 | 14.055296 | 9.981083 | 6.703537 | 9.488555 | Normal |
| *ENSG00000155629* | ***PIK3AP1*** | 2.099306 | 2.144738 | 2.332148 | 3.097745 | 2.37237 | 2.757427 | Normal |
| *ENSG00000166971* | ***AKTIP*** | 3.672518 | 3.315401 | 3.189774 | 4.814967 | 5.130173 | 5.436265 | Normal |
| *ENSG00000117020* | ***AKT3*** | 0.006307 | 0.004968 | 0.010235 | 0.001188 | 0.027826 | 0.010326 | Normal |
| *ENSG00000105221* | ***AKT2*** | 35.854825 | 31.194848 | 30.806291 | 33.062122 | 34.780927 | 33.268338 | Normal |
| *ENSG00000142208* | ***AKT1*** | 26.106424 | 22.753153 | 25.645345 | 25.963688 | 35.953599 | 29.370829 | Normal |
| *ENSG00000198793* | ***MTOR*** | 17.691648 | 17.891162 | 16.607501 | 14.48706 | 17.736047 | 16.393157 | Normal |
| *ENSG00000133703* | ***KRAS*** | 19.022664 | 17.720603 | 17.340586 | 22.04004 | 16.798707 | 20.263999 | Normal |
| *ENSG00000213281* | ***NRAS*** | 26.153879 | 27.448221 | 27.194269 | 21.908609 | 17.296589 | 21.847574 | Normal |
| *ENSG00000174775* | ***HRAS*** | 9.82629 | 10.073067 | 11.126193 | 7.388674 | 11.801028 | 9.188931 | Normal |
| *ENSG00000178764* | ***RAF*** | 14.301676 | 14.551504 | 13.546754 | 11.80744 | 12.198169 | 11.752332 | Normal |
| *ENSG00000132155* | ***RAF1*** | 66.625246 | 69.307376 | 71.611478 | 64.050602 | 71.760657 | 66.403445 | Normal |
| *ENSG00000157764* | ***BRAF*** | 8.938613 | 8.801879 | 8.542814 | 10.76769 | 8.825402 | 10.586104 | Normal |
| *ENSG00000078061* | ***ARAF*** | 12.327015 | 11.668438 | 10.478802 | 11.525636 | 13.844943 | 13.280836 | Normal |
| *ENSG00000076984* | ***MEK*** | 18.020347 | 18.064487 | 15.65832 | 19.302216 | 19.104422 | 18.172459 | Normal |
| *ENSG00000100030* | ***ERK1/2*** | 16.812165 | 16.969084 | 15.967183 | 17.942025 | 17.9946 | 17.55088 | Normal |
